# Molecular Characterization and Clinical Description of Non-Polio Enteroviruses Detected in Stool Samples from HIV-Positive and HIV-Negative Adults in Ghana

**DOI:** 10.3390/v12020221

**Published:** 2020-02-16

**Authors:** Veronica Di Cristanziano, Kristina Weimer, Sindy Böttcher, Fred Stephen Sarfo, Albert Dompreh, Lucio-Garcia Cesar, Elena Knops, Eva Heger, Maike Wirtz, Rolf Kaiser, Betty Norman, Richard Odame Phillips, Torsten Feldt, Kirsten Alexandra Eberhardt

**Affiliations:** 1 Institute of Virology, University of Cologne, Faculty of Medicine and University Hospital of Cologne, 50935 Cologne, Germany; veronica.di-cristanziano@uk-koeln.de (V.D.C.); kristina.weimer@yahoo.de (K.W.); elena.knops@uk-koeln.de (E.K.); eva.heger@uk-koeln.de (E.H.); maike.wirtz@uk-koeln.de (M.W.); rolf.kaiser@uk-koeln.de (R.K.); 2 National Reference Centre for Poliomyelitis and Enteroviruses, Robert Koch Institute, 13353 Berlin, Germany; BoettcherS@rki.de; 3 Kwame Nkrumah University of Science and Technology, Kumasi 00233, Ghana; stephensarfo78@gmail.com (F.S.S.); branorman@yahoo.com (B.N.); rodamephillips@gmail.com (R.O.P.); 4 Komfo Anokye Teaching Hospital, Kumasi 00233, Ghana; adompreh@gmail.com; 5 Autonomous University of the Mexico State, 50000 Toluca, Mexico; cesar_lucio@msn.com; 6 Kumasi Center for Collaborative Research in Tropical Medicine, Kumasi 00233, Ghana; 7 Clinic of Gastroenterology, Hepatology and Infectious Diseases, University Hospital Düsseldorf, 40225 Düsseldorf, Germany; Torsten.Feldt@med.uni-duesseldorf.de; 8 Department of Tropical Medicine, Bernhard Nocht Institute for Tropical Medicine and I. Department of Medicine, University Medical Center Hamburg-Eppendorf, 20359 Hamburg, Germany

**Keywords:** enterovirus, HIV, Ghana, cosavirus, enteric infection

## Abstract

In the post-polio eradication era, increasing attention is given to non-polio enteroviruses. Most of the data about enteroviruses in sub-Saharan Africa are related to acute flaccid paralysis surveillance and target the pediatric population. This study aimed to investigate the presence of enterovirus in PLHIV (people living with HIV) and HIV-negative individuals in Ghana. Stool samples from HIV-positive individuals (*n* = 250) and healthy blood donors (*n* = 102) attending the Komfo Anokye Teaching Hospital in Kumasi, Ghana, were screened by real-time PCR for enterovirus. Molecular typing of the VP1 region was performed. Enterovirus-positive samples were tested for norovirus, adenovirus, rotavirus, sapovirus, and cosaviruses. Twenty-six out of 250 HIV-positive subjects (10.4%) and 14 out of 102 HIV-negative individuals (13.7%) were detected enterovirus-positive, not showing a significant different infection rate between the two groups. HIV-negative individuals were infected with *Enterovirus C* strains only. HIV-positive participants were detected positive for species *Enterovirus A*, *Enterovirus B*, and *Enterovirus C*. Co-infections with other viral enteric pathogens were almost exclusively detected among HIV-positive participants. Overall, the present study provides the first data about enteroviruses within HIV-positive and HIV-negative adults living in Ghana.

## 1. Introduction

Enteroviruses belong to the family *Picornaviridae* and are non-enveloped single stranded RNA viruses. So far more than 100 different serotypes have been identified to infect humans and grouped into 4 species (*Enterovirus A- Enterovirus D*) on the basis of their genetic divergence [1]. Enteroviruses are endemic worldwide and mainly transmitted via the fecal–oral route or by droplets and direct contacts [2]. The most known members of the genus *Enterovirus* are represented by the three poliovirus types (PV), assigned within the species *Enterovirus C*. Polioviruses are the etiologic agents of paralytic poliomyelitis, an infectious disease characterized by acute flaccid paralysis (AFP) and whose main target is children under 5 years of age [3]. The success of the Global Polio Eradication Initiative (GPEI) has dramatically reduced the circulation of wild PVs [4]. Since 1988, the number of polio endemic countries has declined from 125 to 3, represented by Nigeria, Pakistan and Afghanistan, with Nigeria being on the verge of being certified as polio free [4,5].

In the post-polio eradication era, increasing attention is given worldwide to the detection and characterization of non-polio enteroviruses [6,7]. Although enterovirus infections are mostly asymptomatic, the available data evidenced a high pathogenic potential. Different non-polio enteroviruses have been isolated from patients affected from AFP in the context of polio surveillance [6]. Other severe clinical forms associated with enteroviruses include aseptic meningitis, myocarditis, encephalitis, and respiratory diseases [8,9]. Age, gender, and host immune status can influence the clinical presentation and severity of infection [10]. Furthermore, emerging enterovirus variants have been recognized worldwide as causes of severe clinical manifestations in both sporadic and epidemic cases [11,12,13,14,15,16,17,18]. 

In Europe, the non-polio enterovirus network (ENPEN) was recently established, with the aim to collect data on severe enterovirus infections and monitor the circulation of enterovirus types [19]. 

In sub-Saharan Africa, the limited data about enterovirus infections are mostly related to AFP surveillance [20,21,22]. A previous survey in Côte d’Ivoire evidenced a high variety of enterovirus strains circulating in apparently healthy individuals [23]. A high diversity in enterovirus isolates infecting healthy individuals was also observed in other African countries [24,25].

Malnutrition and inadequate supply of water, sanitation, and hygiene presumably play a crucial role in the spread of this group of viruses [26]. However, few data are available about the presence of enteroviruses in immunocompromised individuals living in these areas, namely PLHIV (people living with Human Immunodeficiency Virus). Chronic meningoencephalitis associated to enterovirus in immunocompromised patients have long been known [27,28,29]. Nevertheless, the potential role of HIV-positive patients as a source of enterovirus infections in tropical areas has been barely examined [30]. 

As known, the depletion of CD4+ T lymphocytes and the associated impairment of the immune system make HIV patients more vulnerable to acquiring other infections and to developing more severe clinical manifestations [31]. Remarkably, co-infections with enteric parasitic infections are reported to worsen the progression of the HIV infection to Acquired Immunodeficiency Syndrome (AIDS) [32]. On the contrary, a potential beneficial immunomodulatory effect was evidenced in case of co-infection with *Helicobacter pylori* in HIV-positive persons [33,34]. The impact of enteric viruses in HIV-positive people living in low-income countries is poorly investigated; however, they are considered a relevant cause of morbidity in this group of patients [35]. Furthermore, HIV progression was associated with the expansion of enteric virome [36,37]. 

In Ghana, 300,000 people are estimated to be living with HIV/AIDS and only 70,000 of them are reported to have access to antiretroviral therapy (ART) [38].

The aim of the present study was to analyze and molecularly characterize the spectrum of enteroviruses infecting HIV-positive and HIV-negative adults living in Ghana, in order to evidence if HIV infection was associated with a higher risk for infection with enteroviruses.

## 2. Materials and Methods 

### 2.1. Study Participants

The present study comprised participants from an observational cohort study on clinical and sociodemographic determinants of *H. pylori* infection among HIV-infected and non-infected persons [33,34]. Adult HIV-positive patients from the HIV Outpatient Department of the Komfo Anokye Teaching Hospital in Kumasi, Ghana, were enrolled between November 2011 and November 2012. Additionally, blood donors from the same hospital were recruited to serve as a HIV-negative control group. From this cohort, we randomly selected HIV-positive patients with CD4+ T cell counts below or more than 200 cells/μL (*n*= 119 and 131, respectively), and 102 HIV-negative individuals to analyze the prevalence and clinical implications of human enteroviruses.

### 2.2. Ethics Statement

Ethical approval for this study was obtained from the Committee on Human Research of the Kwame Nkrumah University of Science and Technology in Kumasi, Ghana: HRPE/AP/12/11, and the ethics committee of the Medical Council in Hamburg, Germany: PV3771. Written informed consent was obtained from all participants before enrolment in accordance with the World Medical Association’s Declaration of Helsinki.

### 2.3. Specimen Collection, Handling and Storage

Trained study personnel collected demographic and clinical data using a standardized questionnaire. Aliquots of native stool samples were freshly frozen and stored at −80 °C before being transported to Germany on dry ice. Blood samples were collected and the analysis of CD4+ T cell count was performed in Ghana using a FACSCalibur flow cytometer (Becton Dickinson, Mountain View, California).

### 2.4. Viral Nucleic Acid Extraction 

A 10% suspension of each stool sample was prepared using PBS. Viral nucleic acids were extracted from 700 µL of the stool suspension by using the automated platform VERSANT kPCR Molecular System and the VERSANT Sample Preparation 1.0 Reagents Kit (Siemens Healthcare Diagnostics, Erlangen, Germany) into an elution volume of 100 µl, according to the manufacturer’s instructions.

### 2.5. Detection of Enteroviruses and Other Viral Enteric Co-Infections

Samples were screened for enteroviruses using the Enterovirus real-time RT-PCR Kit (TaqMan) (AnDiaTec GmbH & Co.KG, Kornwestheim, Germany) according to the manufacturer’s protocol. Enterovirus-positive samples were screened for the presence of other viral enteric pathogens. Norovirus (NoV), adenovirus (AdV), rotavirus (RV), astrovirus (HAstV), and sapovirus (SaV) were detected using the multiplex FTD Viral gastroenteritis (Fast-Track Diagnostics, Luxembourg), in accordance with the manufacturer’s instructions. Cosaviruses (CoSV) were detected as described by Kapoor et al. [39]. 

### 2.6. VP1 Amplification, Sequencing and Molecular Typing

All enterovirus strains were sequenced in the 5′ non-coding region (5′NCR) for enterovirus species assignment. Molecular typing of enterovirus strains was performed using generic and species-specific VP1-primer systems [23,25]. Sequencing was done directly on amplification products using BigDye 3.1 (Applied Biosystems). Assignment to enterovirus types was done using BLAST and the Enterovirus Genotyping Tool [40,41]. Sequences were deposited in GenBank under accession numbers MN812172-MN812201.

In addition to molecular assays, enterovirus-positive stool samples were inoculated to RD-A cells and passaged three times. If CPE was observed, RNA was extracted from 140 µL cell culture supernatant and subjected to enterovirus PCR, targeting the 5′ non-coding region (NCR) and VP1 region. Sequencing was performed to exclude the presence of polioviruses.

### 2.7. Statistical Analysis

Statistical analyses were conducted using R (version 3.6.1, R Foundation for Statistical Computing, Vienna, Austria). Categorical variables were compared using either the χ2 test or the Fisher exact test, as appropriate. Continuous variables were expressed as median (interquartile range, IQR) or mean ± standard deviation (SD) and compared using the Wilcoxon rank sum test or the unpaired Student’s t-test. A multiple linear regression model was used to assess the association between covariates and the continuous outcome CD4+ T cell count cells/μL. Two-sided *p*-values were presented, and statistical significance was determined at α = 5%. 

## 3. Results

### 3.1. Characteristics of Enterovirus-positive and Negative Participants

**Figure 1:**
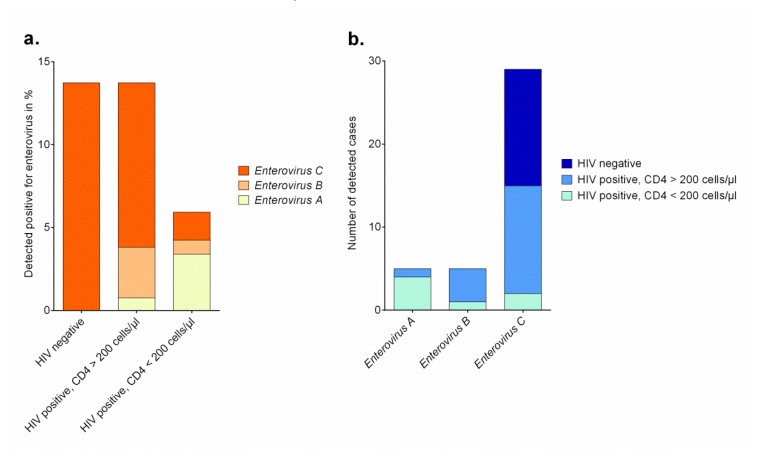
(**a**)**.** Absolute numbers of enterovirus strains assigned to species in HIV-positive adults with high and low CD4+ T cell count and HIV-negative persons in Ghana in %. (**b**)**.** Absolute numbers of enterovirus strains assigned to species detected in adult participants in Ghana.

Forty (11.4%) out of 352 stool samples were tested positive for enteroviruses (Table 1). Out of 250 samples of HIV-positive subjects, twenty-six (10.4%) were tested enterovirus-positive, and fourteen out of 102 HIV-negative individuals (13.7%, *p* = 0.362) were tested enterovirus-positive (Figure 1a). The proportion of HIV infections was not different between enterovirus-positive and enterovirus-negative participants (65.0% vs. 71.8%, respectively). Furthermore, the two groups did not differ regarding the presence of clinical symptoms between enterovirus-positive and enterovirus-negative individuals. Remarkably, within the group of HIV-positive participants, those with an enterovirus infection had significantly higher median CD4+ T cell counts compared to those that were tested enterovirus-negative (416 [IQR 168-535] vs 204 [IQR 78-460] cells/μL, *p* = 0.017), but did not differ in regard to antiretroviral therapy, intake of antibiotics, or time since diagnosis of HIV (Table S1 and S2). Interestingly, this finding was restricted to HIV-positive subjects and was not observed in participants without HIV infection. On the other hand, HIV-negative adults infected with enterovirus were significantly younger than those without enterovirus infection (28.6 ± 6.4 vs. 33.6 ± 12.9, *p* = 0.029).

### 3.2. Enterovirus Typing

Enterovirus strains were characterized by sequencing the V1 region for type assignment. For strains remaining negative for VP1 amplification, sequencing of the 5′ 5′NCR allowed enterovirus species assignment. Thirty-three strains could be assigned to a type and six could be typed on species level. One real-time RT-PCR positive sample remained negative in the typing PCR assays. The identified enterovirus types were assigned to species *Enterovirus A*, *Enterovirus B* and *Enterovirus C* (Table 2). The presence of polioviruses was also excluded using cell culture assays. Only one stool sample (HP0932) showed CPE in RD-A cells and molecular typing resulted in EV-A76 detection. Remarkably, whereas HIV-negative individuals were infected exclusively with *Enterovirus C* strains, HIV-positive participants also showed infections with *Enterovirus A* strains and *Enterovirus B* strains (Figure 1a,b). None of the 15 HIV-infected participants with *Enterovirus C* strains detected reported any gastrointestinal complaints and thirteen out of them had CD4+ T cell counts higher than 200 cells/μL (Table 2). In contrast to this, four out of five participants with *Enterovirus A* strain co-infection had CD4+ T cell counts below 200 cells/μL.

### 3.3. Co-Infection with Other Enteric Viruses

Fifteen (37.5%) out of 40 enterovirus-positive participants were co-infected with additional enteric viruses. Out of them, 10 samples were tested positive for one further viral pathogen and 5 samples for 2 other viruses. AdV and CoSV were the most common co-infections (Table 2). Remarkably, all but one of these infections with additional enteric pathogens were observed in HIV-positive individuals. It is worth noting that four of five co-infections with CoSV in HIV-positive participants were detected in subjects with CD4+ T cell counts below 200 cells/μL (*p* = 0.02).

### 3.4. Factors Associated with CD4+ T Cell Count in HIV-Positive Participants With and Without Enterovirus Co-Infection

In the simple linear regression models age, ART-intake and being infected with Enterovirus C strains compared to not carrying enteroviruses were significantly associated with an increased CD4+ T cell count in HIV-positive participants (Table 3). When adjusting for age, sex and ART-intake, detection of *Enterovirus C* strains was still found to be independently associated with a higher CD4+ T cell count in HIV-positive participants compared to those with an enterovirus-negative status (*p* = 0.042).

## 4. Discussion

Non-polio enteroviruses are recognized worldwide as an emerging cause of different diseases. The outbreaks caused by enteroviruses that affected Europe, America and Asia in the recent years evidenced the high pathogenic potential of this group of viruses [42]. 

Data on enterovirus infections circulating in people living in sub-Saharan Africa are still limited and mostly addressing the pediatric population. Recently, Brouwer et al. detected enteroviruses by real-time RT-PCR in 89.9% of fecal samples collected from a cohort of children ≤ 5 years of age in Malawi, not showing differences in enterovirus detection rates between children with and without severe anemia [43]. So far, no other studies could evidence a comparable high detection rate [44]. 

In our present cohort 40 out of 352 stool samples were tested positive for enteroviruses, indicating a detection rate of 11.4%. Although apparently modest, this result confirms that enterovirus circulation in Ghana is a phenomenon not limited to childhood, considering that the mean age of participants was 38.2 (± 10.6). Nevertheless, we observed a lower mean age in HIV-negative participants infected with enterovirus compared to those without enterovirus infection in our adult cohort. A recent study reported a similar infection rate in healthy individuals living in Nigeria, including children and adults [25]. Apparently, HIV infection was not shown to be associated with a higher rate of enterovirus infections compared to HIV-negative adults. Previous available data seem to confirm this result regardless of age [45,46]. 

However, the higher diversity of enteroviruses as well as the detection of rare and zoonotic enterovirus types, mostly in HIV-positive individuals, provide further incentives to understand better the meaning of enterovirus infections within PLHIV. 

In our study, the majority of strains were assigned to *Enterovirus C* (29/39), which is often described as the most common group circulating in sub-Saharan Africa [43,47]. Within this species, CVA20 and EV-C99 were the most common serotypes identified in our cohort. Although both serotypes have been isolated from AFP patients, they have been more commonly identified in apparently healthy individuals [23,25,43,48]. In our cohort, all but one of the HIV-positive individuals infected with these serotypes had a CD4+ T cell count higher than 200 cells/μL. These two serotypes could be detected in both HIV-positive and HIV-negative individuals. On the contrary, the rarely reported EV-C113 was detected in HIV-positive patients only [49]. 

Interestingly, the detection of enterovirus strains belonging to the species *Enterovirus A* and *Enterovirus B* was restricted to HIV-infected participants. In particular, two HIV-positive participants with CD4+ T cell count > 200 cells/µL were detected positive for two rarely described serotypes, namely EV-B106 and EV-B74 [50,51].

The serotype EV-A119 was detected in three female HIV-positive individuals with CD4+ T cell counts below 200 cells/μL and an age of 45 years or higher, whereas EV-A76 was found in a HIV-positive patient with a low CD4+ T cell count and multiple clinical complaints, including cough, gastrointestinal symptoms, and skin rush. Both of them are known for their zoonotic potential and have been isolated in non-human primates in West and Central Africa [52,53,54,55]. 

The observation that in our cohort only PLHIV were infected with rarely circulating serotypes represents a novel evidence whose meaning needs to be investigated further. The impairment of gastrointestinal immunity induced by HIV might be one possible explanation. 

In line with this result, infections with other enteric viral pathogens in addition to infection with enteroviruses could be found almost exclusively in HIV-positive patients. 

It is interesting to note that the detection of CoSV infections in enterovirus-positive individuals (6/40, 15%) was rather higher when compared to the limited available data of other cohorts of adults [25]. So far, CoSV infections in adults have been rarely reported worldwide. CoSV were found in one of 1000 adult patients with gastroenteritis in Scotland and in one of 150 stool samples from adults with diarrhea living in Thailand [39,56]. Similar data were obtained from immunocompromised individuals. Among HIV-infected patients, CoSV were detected in one of 154 patients suffering from gastroenteritis in Brazil and in three of 196 patients with and without diarrhea in the Netherlands [57,58]. A case of persistent CoSV infection and chronic diarrhea was also described in a lung transplant recipient [59]. 

In the present cohort, the almost exclusive detection of other viral enteric agents in enterovirus-positive persons infected with HIV is suggestive of an altered intestinal mucosal immunity affecting this group of patients.

A recent investigation targeting a cohort of Ugandan patients evidenced that advanced HIV/AIDS is associated with the expansion of enteric virome, in particular adenoviruses [36]. Our data confirmed a higher burden, in terms of enterovirus variability and rate of co-infections with other gastrointestinal viruses, among HIV-infected patients compared to HIV-negative individuals. Adenovirus represented the most common viral agent detected as co-infection. 

The association of the CD4+ T cell count in HIV-positive individuals with different enterovirus species represents a new aspect of the present study. Rarely reported *Enterovirus A* infections as well as infections with zoonotic potential types were almost exclusively detected in HIV-positive individuals with an impaired immune system, as indicated by a low CD4+ T cell count.

Contrastingly, the infection with members of *Enterovirus C* types in HIV-positive participants was independently associated with a higher CD4+ T cell count compared to PLHIV not infected with enteroviruses. So far, *Enterovirus C* is the only species of human enteroviruses that was not detected in animals, confirming it as highly specific to humans. Considering that *Enterovirus C* species is more prevalent in healthy people, it might be supposed that members of this group of viruses are able to stably replicate in the human gut, reflecting a favorable health status [60]. On the other hand, the higher CD4+ T cells/μL count detected in HIV+ individuals infected with *Enterovirus C* strains compared to HIV-positive participants without enterovirus infection might indicate a cell-mediated immune response toward the co-infection.

Another remarkable aspect of this study is represented by the fact that co-infection with CoSV was significantly more common in enterovirus-infected PLHIV with CD4+ T cell counts below 200 cells/μL, indicating the potential role of CoSV as an opportunistic agent. 

Overall, the present study provides the first data about enteroviruses and additional enteric co-infections within HIV-positive and HIV-negative adults living in Ghana. Although infections with enteroviruses were not more frequent in PLHIV, a higher diversity of enteroviruses, including rare and zoonotic types, was predominantly detected among HIV-positive individuals with a low CD4+T cell count. Infections with additional enteric viruses were almost exclusively detected among HIV-infected individuals, confirming the detrimental effect of HIV on gut homeostasis. As prolonged enterovirus shedding might be a biomarker for defective immune defenses against enteroviruses, it would be of interest if HIV-positive individuals infected with enterovirus had prolonged shedding of the same serotype in consecutive stool samples. Therefore, further longitudinal studies are required to gain more comprehensive knowledge about the role of such viruses on gut homeostasis.

Surveillance of non-polio enterovirus diversity among distinct patient groups in African countries, including PLHIV, plays an important role in not underestimating this neglected occurrence of EV infections and their potential impact on public health.

## Figures and Tables

**Table 1:** Demographical and clinical characteristics of enterovirus-positive and enterovirus-negative participants.

Parameters	Total (*n* = 352)	Enterovirus-Positive Subjects (*n* = 40, 11.4%)	Enterovirus-Negative Subjects (*n* = 312, 88.6%)	*p*-Value
Age in years, mean ± SD:	38.2 ± 10.6	37.7 ± 10.7	38.3 ± 10.6	0.760
in HIV-positive subjects	40.3 ± 9.1	42.3 ± 9.2	40.0 ± 9.1	0.186
in HIV-negative subjects	32.9 ± 12.3	28.6 ± 6.4	33.6 ± 12.9	0.029
Female gender, *n* (%)	247 (70.6)	31 (77.5)	216 (69.7)	0.307
HIV status:				
positive, *n* (%)	250 (71.0)	26 (65.0)	224 (71.8)	0.372
negative, *n* (%)	102 (29.0)	14 (35.0)	88 (28.2)	
CD4+ T cells/μL (IQR):				
in HIV-positive subjects	221 (87–474)	416 (168–535)	204 (78–460)	0.017
in HIV-negative subjects	969 (786–1150)	958 (769–1038)	982 (787–1153)	0.726
Low BMI (<18.5 kg/m²), *n* (%):	37 (11.1)	1 (2.7)	36 (12.2)	0.099
in HIV-positive subjects	34 (13.9)	1 (3.8)	33 (15.1)	0.143
in HIV-negative subjects	3 (3.4)	0 (0.0)	3 (3.9)	1.00
Clinical symptoms during last 6 months:				
Gastrointestinal symptoms, *n* (%)	64 (18.4)	8 (20.0)	56 (18.2)	0.780
Feverish symptoms, *n* (%)	42 (18.5)	5 (18.5)	37 (18.5)	0.998
Cough, *n* (%)	56 (16.1)	7 (17.5)	49 (15.9)	0.797
Skin rashes, *n* (%)	26 (7.5)	3 (7.5)	23 (7.5)	1.000
Access to tap water, *n* (%)	190 (54.6)	21 (52.5)	169 (54.9)	0.777
Fridge/freezer in household, *n* (%)	249 (71.6)	30 (75.0)	219 (71.1)	0.607
Electricity in household, *n* (%)	326 (93.7)	40 (100.0)	286 (92.9)	0.091

IQR—interquartile range; SD—standard deviation; BMI—Body Mass Index.

**Table 2:** Characteristics, clinical features and enterovirus typing results of enterovirus-positive adults living in Ghana.

ID	HIV Status	Sex	Age in Years	CD4+ T Cells/μl	On ART	Feverish Symptoms	Cough	Gastrointestinal Symptoms	Skin Rashes	Enterovirus Typing Result	Enterovirus Species	Other Enteric Infections
CHP012	neg	f	33	NA	NA	-	-	-	-	*Enterovirus C*	*Enterovirus C*	-
CHP013	neg	f	23	1276	NA	-	-	-	-	CVA19	*Enterovirus C*	-
CHP021	neg	f	32	921	NA	-	-	-	-	CVA13	*Enterovirus C*	-
CHP023	neg	f	28	940	NA	-	-	+	-	*Enterovirus C*	*Enterovirus C*	-
CHP027	neg	m	23	977	NA	-	-	-	-	CVA20	*Enterovirus C*	-
CHP028	neg	f	29	735	NA	+	+	-	-	CVA13	*Enterovirus C*	-
CHP029	neg	f	26	1698	NA	-	-	-	-	EV-C99	*Enterovirus C*	-
CHP042	neg	f	33	967	NA	+	-	+	+	*Enterovirus C*	*Enterovirus C*	-
CHP051	neg	m	19	958	NA	-	-	+	-	CVA19	*Enterovirus C*	-
CHP061	neg	m	22	1381	NA	-	-	-	-	EV-C99	*Enterovirus C*	CoSV-D
CHP065	neg	m	39	680	NA	-	-	-	-	*Enterovirus C*	*Enterovirus C*	-
CHP105	neg	f	25	1038	NA	-	-	+	+	*Enterovirus C*	*Enterovirus C*	-
CHP138	neg	m	27	363	NA	-	-	-	-	CVA20	*Enterovirus C*	-
CHP165	neg	f	41	769	NA	-	+	-	-	EV-C99	*Enterovirus C*	-
HP0008	pos	f	55	65	-	NA	-	+	-	EV-A119	*Enterovirus A*	AdV, CoSV-A
HP0010	pos	m	56	522	+	+	-	-	-	EV-C99	*Enterovirus C*	NoV G2, AdV
HP0038	pos	m	38	396	+	NA	-	-	-	CVA5	*Enterovirus A*	-
HP0063	pos	f	40	470	+	NA	-	-	-	EV-C99	*Enterovirus C*	-
HP0120	pos	f	50	331	-	NA	-	-	-	CVA20	*Enterovirus C*	-
HP0123	pos	f	28	134	-	NA	-	-	-	CVA17	*Enterovirus C*	AdV
HP0130	pos	f	30	465	-	NA	-	-	-	*Enterovirus C*	*Enterovirus C*	-
HP0210	pos	f	48	356	-	+	-	-	-	E27	*Enterovirus B*	NoV GI
HP0216	pos	m	43	851	-	NA	-	-	-	EV-C113	*Enterovirus C*	-
HP0255	pos	f	26	455	-	NA	-	-	-	EV-C113	*Enterovirus C*	-
HP0274	pos	f	43	539	+	NA	+	-	-	CVA20	*Enterovirus C*	AdV
HP0297	pos	f	36	655	+	NA	+	+	-	EV-B74	*Enterovirus B*	SaV
HP0335	pos	f	36	391	+	NA	-	-	-	EV-C113	*Enterovirus C*	SaV
HP0361	pos	f	27	725	+	NA	-	-	-	CVA20	*Enterovirus C*	-
HP0407	pos	f	47	1677	+	NA	-	-	-	CVA19	*Enterovirus C*	-
HP0578	pos	f	59	635	+	-	-	-	-	CVA13	*Enterovirus C*	NoV GII
HP0648	pos	f	57	318	-	-	-	-	-	E14	*Enterovirus B*	-
HP0762	pos	f	45	151	-	-	-	-	-	EV-A119	*Enterovirus A*	AdV, CoSV-D
HP0800	pos	f	44	555	-	+	+	+	-	EV-B106	*Enterovirus B*	AdV
HP0845	pos	m	39	93	-	-	-	-	-	CVB6	*Enterovirus B*	CoSV-D
HP0916	pos	f	38	181	+	-	-	-	-	CVA20	*Enterovirus C*	-
HP0932	pos	f	51	83	-	-	+	+	+	EV-A76	*Enterovirus A*	CoSV-B
HP0995	pos	f	37	435	+	-	+	-	-	CVA13	*Enterovirus C*	SaV, CoSV-D
HP1007	pos	f	47	87	+	-	-	-	-	EV-A119	*Enterovirus A*	NoV GII2, AdV
HP1097	pos	f	40	163	-	-	-	-	-	Positive	NA	-
HP1107	pos	f	48	466	-	-	-	-	-	EV-C99	*Enterovirus C*	-

+—present; -—not present; f—female; m—male.

**Table 3:** Factors associated with increased CD4+ T cell count in HIV-positive participants with and without enterovirus co-infection.

	HIV-Positive With and Without Enterovirus Co-Infection			
	Simple Linear Regression Models		Multiple Linear Regression Model	
Variable	β-Coef	*p* Value	β-Coef	*p* Value
Age in years	4.12	0.077	3.91	0.073
Male	−41.74	0.381	−28.65	0.517
ART intake	275.13	<0.001	262.09	<0.001
Enterovirus species compared to enterovirus-negative status				
*Enterovirus A*	−160.03	0.287	−199.38	0.152
*Enterovirus B*	78.975	0.599	101.43	0.465
*Enterovirus C*	235.37	0.008	167.77	0.042

ART—antiretroviral therapy; β-Coef—linear regression coefficient.

## Supplementary Materials

Supplementary materials can be found at https://www.mdpi.com/1999-4915/12/2/221/s1.
