# Supplementary material for: Molecular Characterization and Clinical Description of Non-Polio Enteroviruses Detected in Stool Samples from HIV-Positive and HIV-Negative Adults in Ghana
Source: Viruses. 2020 Feb 16;12(2):221. doi: 10.3390/v12020221 (PMC7077198; doi:10.3390/v12020221)
Supplement: Supplementary file 1 [file viruses-12-00221-s001.zip › Supplementary Material DiCristanziano et al..docx]

**Supplementary Material**

“Molecular characterization and clinical description of non-polio enteroviruses detected in stool samples from HIV positive and negative adults in Ghana”, Di Cristanziano et al.

Table S1. Comparison of parameters related to HIV-infection between enterovirus positive and negative HIV positive participants.

|  | **HIV positive (n=250)** | |  |
| --- | --- | --- | --- |
| **Parameters** | **Enterovirus positive (n=26)** | **Enterovirus negative (n=224)** | **p-value** |
| Time since diagnosis of HIV infection in months, (IQR) | 14 (0-43) | 0 (0-41) | 0.210 |
| ART intake, n (%) | 12 (46.2) | 82 (36.6) | 0.341 |
| Time since initiation of ART in months, (IQR) | 40 (16-54) | 49 (16-66) | 0.661 |
| Co-trimoxazole intake, n (%) | 10 (38.5) | 66 (29.46) | 0.345 |
| Rifampicin intake, n (%) | 3 (11.5) | 27 (12.1) | 1.000 |
| Intake of other antibiotics, n (%) | 1 (3.8) | 2 (0.9) | 0.282 |

Table S2. CD4+ T cell counts in cells/μl in different age groups in HIV positive participants.

|  | **Kruskal-Wallis test** | | **Simple linear regression model** | |
| --- | --- | --- | --- | --- |
| **Parameters** | **Total (n=250)** | **p-value** | **β-Coef** | **p-value** |
| CD4+ T cell count in cells/μl per age group, (IQR)  < 30 years  30-40 years  41-50 years  > 50 years | 175 (56-530)  203 (93-427)  239 (78-540)  241 (110-463) | 0.807 | 1  -50.97  -0.45  65.15 | 0.440  0.995  0.394 |
